# An Attempted Correlation Between the Fecal Microbial Community of Chinese Forest Musk Deer (*Moschus berezovskii*) and Differences in Musk Production and Quality

**DOI:** 10.3390/ani15111622

**Published:** 2025-05-31

**Authors:** Tingting Zheng, Qian Liu, Chengli Zheng, Xiuxiang Meng, Xue Bai, Diyan Li, Tao Wang, Jun Guo, Zhongxian Xu, Hang Jie

**Affiliations:** 1 Nanchong Key Laboratory of Wildlife Nutrition Ecology and Disease Control, Key Laboratory of Southwest China Wildlife Resources Conservation (Ministry of Education), China West Normal University, Nanchong 637002, China; ztt12172024@163.com (T.Z.); qianliu0908@163.com (Q.L.); is_guojun@163.com (J.G.); 2 Jinfo Mountain Forest Ecosystem Field Scientific Observation and Research Station of Chongqing, Chongqing Institute of Medicinal Plant Cultivation, Nanchuan, Chongqing 404100, China; 3 Sichuan Institute of Musk Deer Breeding, Sichuan Institute for Drug Control, Chengdu 610106, China; zhengcl@scidc.org.cn; 4 School of Resources and Environment, Aba Teachers University, Aba 623002, China; meng2014@ruc.edu.cn; 5 School of Pharmacy, Chengdu University, Chengdu 610106, China; 18792972819@163.com (X.B.); lidiyan@cdu.edu.cn (D.L.); 6 College of Basic Medicine, Chengdu University, Chengdu 610106, China; wanttao@hotmail.com

**Keywords:** musk, fecal fungi, ITS, musk production, musk quality

## Abstract

Musk, a dried secretion from the sac gland near the urethral foramen of adult male forest musk deer (*Moschus berezovskii*), has significant economic value and is extensively utilized as a valuable component in traditional Chinese medicine. In the practice of forest musk deer breeding, musk with different colors and varying moisture contents is observed during the season when the musk reaches maturity. For many years, researchers have focused mainly on musk composition and symbiotic bacteria. However, the influence of fecal fungi on the production and quality of musk is unknown. In this study, internal transcribed spacer (ITS) analysis was employed to explore the relationships between the fungal composition of musk deer fecal and the quality and production of musk produced by each individual. The results indicate that fungal genera known to cause diseases, such as *Colletotrichum* and *Apiotrichum*, are prevalent in the feces of musk deer that produce abnormal musk. Furthermore, the fecal microbiota health index (GMHI) is lower and the intestinal microbiota dysbiosis index (MDI) is greater in musk deer producing white musk than in normal individuals. Additionally, by correlating musk production with fecal fungi, we also found that *Dolichousnea* and *Scolecoleotia* were significantly positively correlated with musk production. Moreover, *Metschnikowia*, *Ganodermataceae_gen_Incertae_sedis*, *Hypoxylon*, *Neovaginatispora*, *Didymella*, *Dothidea*, and *Trichoderma* were negatively correlated with musk production. This study is the first to investigate gut fungi in relation to musk production/quality, establish gut health and fungal dysbiosis links, and identify candidate fungi tightly associated with musk traits. This exploratory approach is critical for exploring uncharted territories like gut fungi in musk deer and musk traits.

## Simple Summary

Musk, a valuable glandular secretion from male forest musk deer, is critical for traditional medicine and the perfume industry. While prior research has focused on musk composition and bacteria, the role of fecal fungi in influencing musk production and quality remains unknown. This study, the first to explore this uncharted area, analyzed fecal fungal communities using ITS sequencing in 89 musk deer, linking fungal profiles to musk traits like color, moisture, and yield. Key findings reveal that pathogenic fungi, such as *Colletotrichum* and *Apiotrichum*, were abundant in deer producing abnormal musk (e.g., white or mud-like), which correlated with lower GMHI and MDI. Conversely, the genera *Dolichousnea* and *Scolecoleotia* were significantly associated with increased musk production. This exploratory research establishes foundational links between gut fungal imbalance and abnormal musk production, highlighting potential beneficial fungi for improving musk yield.

## 1. Introduction

The forest musk deer (*Moschus berezovskii)* is a rare and endangered animal in China. It is economically important and now protected at the national level [1,2]. Musk, which is secreted by adult male *M. berezovskii*, is not only an essential component in traditional Chinese medicine but also has extensive applications in the perfume industry [3]. Populations of wild *M. berezovskii* have declined dramatically due to the high demand for musk [4]. To curb this trend and meet the demand for musk, China has reared *M. berezovskii* in captivity since 1958. The musk sac is an organ that synthesizes, stores, and secretes musk. In an adult male forest musk deer, it is located between its navel and genitals. The formation of musk is a lengthy process that takes several months. Firstly, the initial musk, which is in a liquid state and has a faint yellow color, is secreted by musk gland cells [2,5,6]. Subsequently, the initial musk enters the musk sac through the duct, and is finally stored and matured in the musk sac. During this process, the moisture content, color, and composition of the initial musk gradually change from a viscous cream-colored liquid with a fishy odor to a blackish-brown solid substance with a strong fragrance upon full maturation [5,6,7]. Under normal circumstances, healthy adult males eventually form solid substances that are black or brown in color [6,7]. These emit a potent fragrance with a rich, oily texture and are regarded as the highest-quality musk [8,9]. However, in actual breeding populations of forest musk deer, two types of abnormal musk have been discovered: one is white with a sour and rotten smell, and the other is mud-like with an unpleasant foul odor [10]. Although parasitosis, diarrhea [11], and abscesses [12] are common diseases that plague the health of musk deer, the individuals secreting white musk may suffer from chronic stress and more diseases [10].

The fecal microbiota plays a crucial role in the body’s metabolism and health regulation [13,14]. There are two major types of microbes (bacteria and fungi) that live symbiotically with their hosts. Studies have been carried out on the effects of symbiotic bacteria in feces [15] and the musk sac [7,16] can affect the health [17] and musk production of deer [18]. Currently, there have been no studies on the impact of fecal fungi in forest musk deer on their musk production, but we cannot overlook the significance of fungi. As a fungi, *Saccharomyces cerevisiae* has great potential in increasing the production of ketones, such as 2-nonanone, 2-undecanone, 2-tridecanone, and 2-pentadecanone through its inherent peroxisomal fatty acid β-oxidation cycle [19]. *Saccharomyces boulardii* has been widely used as probiotic fungi to treat the diarrhea symptoms of irritable bowel syndrome [20]. *Aspergillus* produces polysaccharides, which in turn affect the host’s intestinal absorption and metabolism of short-chain fatty acids and bile acids [21]. *Candida* participates in glycolysis, fatty acid, and amino acid metabolism [22]. This indicates that fatty acids, amino acids, and ketones that are metabolized by these fecal fungi are probably sources of active components in musk. Therefore, we speculated that fungi in the feces of forest musk deer may also contribute to the synthesis of musk by forming a gut–gland axis where microbial metabolites modulate glandular cell activity.

The ITS is part of the non-transcriptional region of the fungal rRNA gene, located between the small and large subunits, and contains the fastest-evolving sequences [23]. ITS sequencing, with its high resolution, ease of operation, and broad applicability, has become an essential tool for fungal taxonomy and diversity studies. It is widely used in environmental, medical [24], plant, animal [25,26], and food fungi [27] studies to analyze their classification, development, and functions [28]. Here, we focus on the effects of fecal fungi on the production and quality of musk. We collected 89 fresh fecal samples, recorded the production and quality traits (color and moisture contents) of mature musk.We utilized the latest fungal sequencing technology (ITS sequencing) to investigate the effects of fecal fungi on the production and different qualities of musk in forest musk deer. This study may be helpful for providing a theoretical basis for the increase in musk production and quality in forest musk deer.

## 2. Material and Methods

### 2.1. Animals and Sample Collection

In this study, the fecal fungi of forest musk deer were sourced from the Forest Musk Deer Breeding Farm in Maerkang, Sichuan Province. During the feeding process of forest musk deer, their diet, behavior, feces, and the physiological conditions of the musk gland were monitored at all times. The dietary standardization and quality control of all musk deer were strictly managed, as described in our previous study [29]. The breeding farm regularly screens the deer for pathogens, including *Escherichia coli*, *Clostridium perfringens*, *Brucella*, and *Clostridium tetani*. Feces, which are non-invasive samples [30] and ubiquitously used as representatives for the gut microbiome population in rare animals [31] and humans [30], were collected in this study. They were taken from individuals that had not been administered any antibiotics or anti-inflammatory drugs within the previous six months, and which had not suffered any injuries, ensuring that the sampled individuals were healthy. Each forest musk deer was raised under the same feeding requirements and standards as the breeding farm, with free access to water and maintained at an appropriate living temperature and humidity. Male musk deer were housed individually in pens of ~35 m^2^ for indoor feeding and rest, with access to an outdoor exercise area of 150–200 m^2^ per individual (with a total outdoor exercise area of 15–20 Km^2^). They spent about 4 h a day in the pen feeding and resting, and the rest of the time they were outdoors. During the musk maturation period (October), fecal samples of about 10 g were collected from 89 adult male forest musk deer aged between 4 and 12 years. In order to ensure the freshness of feces, the droppings of every musk deer were collected at 6:00–6:30 in the morning. The collection of musk was conducted according to our previous method [29].We evaluated the appearance of the collected musk (including color assessment and moisture content evaluation), recorded the production of musk (Supplementary Table S1), and also collected feces from the forest musk deer that produced the musk. The collected samples were placed in a sterile bag, immediately frozen in dry ice for transportation, and finally transferred to an ultralow-temperature freezer at −80 °C for storage.

### 2.2. Musk Morphological Characteristics Assessment

Musk color was determined using a standardized visual assessment protocol aligned with industry and institutional guidelines for musk quality evaluation. The color categories (white, brown, black, dark brown, reddish-brown, yellowish-brown) were defined based on the color descriptors for musk quality in traditional Chinese medicine [8]. We employed a validated color grading system using a physical reference panel (printed swatches of standardized color blocks) to ensure consistency across assessments based on captive breeding standards. To reduce inter-observer variability, standardized lighting conditions, dual independent evaluation, and quantitative calibration were employed, and color assessment was conducted by the same trained technicians.

Due to musk scarcity and high economic value, the moisture content was measured in 20 representative samples spanning the 4 predefined groups (powder, paste, strip, mud-like), with 5 samples per group (Supplementary Table S2). The moisture content of musk was determined using a standard gravimetric method to ensure quantitative accuracy, detailed as follows: Approximately 1.0 g of fresh musk was weighed and placed in pre-dried, tared aluminum dishes. The samples were dried in a forced-air oven for 24 h at 105 ± 2 °C, which is a standard temperature for moisture determination in semi-solid biological samples to prevent the thermal degradation of volatile compounds (e.g., musk ketones). The dishes were cooled in a desiccator for 30 min and reweighed until a constant weight was achieved (variation of < 0.001 g between consecutive measurements). The moisture content (%) was calculated using the following formula: Moisture Content (%) = (Initial Weight − Final Dry Weight)/Initial Weight × 100. [Powder: Moisture content < 50% (free-flowing granular texture). Strip: 50–60% moisture (elastic, fibrous consistency). Paste: 60–70% moisture (pliable, non-flowing solid). Mud-like: >70% moisture (viscous, paste-like with high fluidity).

### 2.3. DNA Extraction and PCR Amplification

Total microbial genomic DNA was extracted from the feces of the forest musk deer samples using the YH-Feces Stool DNA Extraction Kit (Yuhua, Shanghai, China) according to the manufacturer’s instructions. The DNA quality and concentration were assessed through 1.0% agarose gel electrophoresis and measured using a NanoDrop 2000 spectrophotometer (Thermo Scientific, Waltham, MA, USA). The samples were then stored at −80 °C for subsequent use. The hypervariable region ITS1F-ITS2R of the fungal ITS gene was amplified with the primer pairs ITS1F (5′-CCGCGGCKGCTGGCAC-3′) and ITS2R (5′-GCTGCGTTCTTCATCGATGC-3′) [32] using a T100 minThermal Cycler Polymerase Chain Reaction (PCR) thermocycler (Bio-Rad, Hercules, USA). The PCR mixture included 4 μL of 5× Fast Pfu buffer, 2 μL of 2.5 mM dNTPs, 0.8 μL of each primer (5 μM), 0.4 μL of Fast Pfu polymerase, 10 ng of template DNA, and ddH_2_O to a final volume of 20 µL. PCR amplification was performed under the following cycling conditions: initial denaturation at 95 °C for 3 min, followed by 27 cycles consisting of denaturation at 95 °C for 30 s, annealing at 55 °C for 30 s, and extension at 72 °C for 45 s. This was followed by a final extension at 72 °C for 10 min, and the reaction was held at 4 °C upon completion. The PCR product was then extracted from a 2% agarose gel and purified using a PCR Clean-Up Kit (YuHua, Shanghai, China) according to the manufacturer’s instructions. The purified product was quantified using a Qubit 4.0 fluorometer (Thermo Fisher Scientific, Waltham, MA, USA). To ensure the accuracy of the experiment, we conducted three replicates of the PCR experiment. A negative control with 10 ng ddH_2_O was used to replace the template DNA.

### 2.4. Illumina Sequencing

Purified amplicons were pooled in equimolar amounts and paired-end sequenced on an Illumina NextSeq 2000 platform (Illumina, San Diego, CA, USA) according to the standard protocols of Majorbio Bio-Pharm Technology Co., Ltd. (Shanghai, China). The raw sequencing reads were deposited into the NCBI Sequence Read Archive (SRA) database (accession number: PRJNA1203064).

### 2.5. Amplicon Sequence Processing and Analysis

Following the demultiplexing process, the sequences underwent quality control using fastp software version 0.19.6 to ensure data integrity [33] and merged using FLASH (v1.2.11) [34]. The high-quality sequences were subsequently denoised using the Divisive Amplicon Denoising Algorithm 2 (DADA2) [35] plugin in the Quantitative Insights Into Microbial Ecology (QIIME) [36] (version 2020.2) pipeline with the recommended parameters, which yielded single-nucleotide resolution based on the error profiles of the samples. DADA2-denoised sequences are usually called amplicon sequence variants (ASVs). To mitigate the impact of sequencing depth on the assessment of alpha and beta diversity, the sequencing reads from each sample were rarefied to a uniform count of 8619. This approach maintained an average Good’s coverage of 97.90%, ensuring robustness in our diversity measurements. Taxonomic classification of the ASVs was subsequently conducted using the naive Bayes consensus taxonomy classifier in the QIIME software package.

### 2.6. Statistical Analysis

The bioinformatic analysis of the fecal microbiota was performed utilizing the Majorbio Cloud platform. Leveraging the ASV data, we computed rarefaction curves and various alpha diversity indices, such as the number of observed ASVs, the Chao1 richness estimator, and Good’s coverage, employing Mothur version 1.30.1 for these calculations [37].

#### 2.6.1. Diversity Analysis

To assess the fungal diversity within a community and their relative abundance. Alpha diversity analysis was conducted after excluding samples with a coverage rate of 97%. Alpha diversity indices (Chao1 indices) were calculated on the rarified dataset using Mothur (v1.30.2). These diversity indices were then compared with the Kruskal‒Wallis test using the stats package in R (v4.0.0).

We used β diversity to evaluate the differences among the samples. The β diversity was calculated in BC and weighted/unweighted UniFrac distances using QIIME2 2023.9 software [36]. The BC ordination provided position values along an ordination axis and distances from the axis for samples of communities.

We conducted principal coordinate analysis (PCoA) to derive principal coordinates and visualize intricate, high-dimensional datasets. The distance matrix, which consisted of the weighted and unweighted UniFrac distances between samples that had been calculated earlier, was restructured into a fresh set of perpendicular axes. The first principal coordinate represented the greatest degree of variation, the second principal coordinate represented the second-greatest degree of variation, and this pattern continued. The similarity among the microbial communities in different samples was determined by PCoA based on the Bray‒Curtis dissimilarity using the Vegan v2.5-3 package.

#### 2.6.2. Fecal Microbiota Health Index (GMHI) and Microbial Dysbiosis Index (MDI) Assessment

Fecal GMHI is a robust index for assessing health status based on species-level taxonomic features of fecal microbiome samples (i.e., the degree of disease presence). It focuses on determining the likelihood of illness and can be used independently for clinical diagnosis. This method is primarily achieved by comparing the relative abundances of microbial species between two groups representing good and poor health conditions [38]. MDI is an index for determining the degree of microbial ecological imbalance. A higher MDI value indicates a greater degree of microbial disruption [39]. The methodologies of GMHI and MDI were calculated following the descriptions of previous studies [38,39].

#### 2.6.3. Community Difference Analysis

To identify the significant differential fungal communities among the different musk yields and quality groups, comparisons of taxonomic data at the phylum and genus levels among different groups were performed using the Kruskal–Wallis test with Tukey’s post hoc HSD test using the stats package in R (v4.0.0). Statistical significance was accepted as *p* < 0.05.

#### 2.6.4. Correlation Heatmap Construction

To investigate presentative fungal that might participate in musk maturation, Spearman correlation heatmaps were generated to estimate the relationships between fungal and musk production at the genus level using the pheatmap package in R (v4.0.0).

#### 2.6.5. Prediction of the Functional Profiles of the Microbial Communities

To parse the fungal community dataset from the rarefied ASV table into functional groups (or guilds), the online resource FUNGuild2024 (http://www.funguild.org/, accessed on 1 December 2024) was used [40]. FUNGuild software was employed to annotate the taxonomic data within the operational taxonomic unit (ASV) table by referencing its online database. This annotation process assigned functional guilds, trophic modes, and growth morphologies to each taxonomic entry. To ensure the accuracy of our annotations, only those with confidence scores of ‘Probable’ and ‘Highly Probable’ were considered for inclusion in our analysis.

## 3. Results

### 3.1. Characteristics and Grouping of Musk Quality

**Figure 1:**
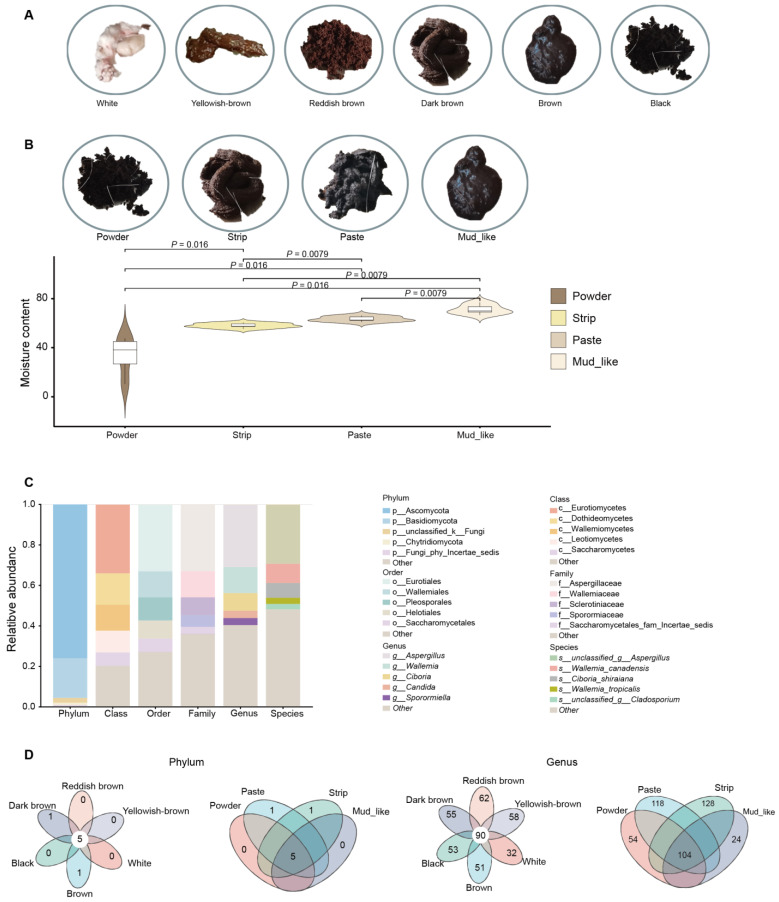
The morphological characteristics of musk and fecal fungal communities in musk deer. (**A**) Images of musk in various colors. (**B**) Images of musk with different moisture contents, and s chart displaying the differences in moisture content. (**C**) The top five relative abundances of various fungi according to species, genus, family, order, class, and phylum. (**D**) Venn diagram illustrating the fungi communities in different groups at the phylum and genus levels in the color and moisture content groups, respectively. The number listed in the center represents the core fungal taxa common to all musk groups, and the number on the petal indicates a unique community for each group.

In this study, a nonsignificant difference was detected between age and musk production, Therefore, the influence of age was not considered (Figure S1). Therefore, we conducted a grouped study on musk yield and quality (Table S1). The correlation analysis was conducted between age, musk quality and production. According to the musk quality factors, the samples were divided into six groups (n = 83) based on color: white (n = 7), brown (n = 17), black (n = 14), dark brown (n = 12), reddish-brown (n = 15), and yellowish-brown (n = 18). The samples were also divided into four groups (n = 83) based on moisture content: powder (n = 13), paste (n = 30), strip (n = 35), and mud-like (n = 5). In addition, six individuals had zero musk production. The colors and moisture contents of the musk are illustrated in Figure 1A,B. A total of 20 samples were selected for moisture content detection, showing significant differences (*p* < 0.05) among the group (Supplementary Table S2, Figure 1B).

### 3.2. Statistics of Sequencing Data

Sequencing of the ITS sequences of the fecal fungus was performed. As a result, a total of 6,617,166 (74,350 reads/sample) and 6,470,826 (72,705 reads/sample) high-quality clean reads were obtained from the ITS-sequenced samples (Supplementary Table S3). Cluster analysis was conducted on the clean reads, and a total of 9768 ASVs were obtained. The rarefaction curves of the Chao1 indices at the ASV level gradually decreased as the sequencing depth increased (Figure S2). The results demonstrate that each fecal sample had sufficient ASVs to reflect the maximum level of fungal diversity, which indicates a sufficient sequencing depth.

In the unite9.0/its_fungi database, for 89 fecal fungal communities, we subsequently classified the 3993 ASVs into 14 phyla, 49 classes, 133 orders, 337 families, 725 genera, and 1266 species. Among these taxa, two phyla and one genus were detected in all the samples. The five phyla with the highest average relative abundances were Ascomycota, Basidiomycota, unclassified_k__Fungi, Chytridiomycota, and Fungi_phy_Incertae_sedis, with corresponding relative abundances of 75.93%, 12.62%, 2.36%, 1.16%, and 0.68%, respectively. Among the 725 genera, the top five genera with relatively high abundances were *Aspergillus*, *Wallemia*, *Ciboria*, *Candida*, and *Sporormiella*, with corresponding relative abundances of 30.94%, 12.83%, 8.72%, 3.62%, and 3.45%, respectively (Figure 1C). At the phylum level, there were five overlapping phyla in both the color and moisture content groups (Figure 1D). At the genus level, 90 genera overlapped among the six color groups, and 104 genera overlapped among the four moisture content groups (Figure 1D).

### 3.3. Fungal Content in Different Musk Color and Moisture Content Groups

**Figure 2:**
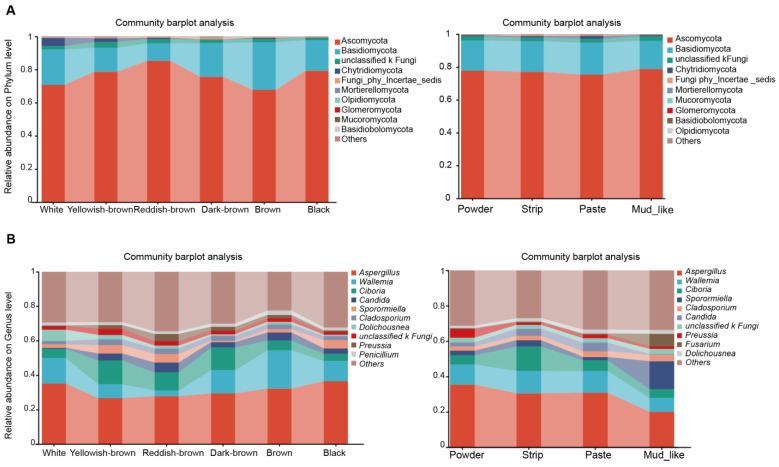
Cumulative barplot charts of fungi communities. The relative abundance of fungi at the phylum (**A**) and genus (**B**) levels in musk samples with different colors and moisture contents.

For the fungal community in the feces, at the phylum level, the results indicate that different groups of microorganisms presented differences. In terms of both color and moisture content, Ascomycota and Basidiomycota were the most prevalent phyla, and the relative abundances of Ascomycota, Basidiomycota, and unclassified Fungi increased with increases in the musk moisture content (Figure 2A). At the genus level, *Aspergillus*, *Wallemia*, and *Ciboria* were the dominant taxa with respect to white, brown, dark brown, and yellowish-brown musk; *Aspergillus*, *Wallemia*, and *Sporormiella* were the dominant genera with respect to black musk; and reddish-brown musk was associated with *Aspergillus*, *Ciboria*, and *Candida* (Figure 2B). The overall trend was that the relative abundance increased as the color intensified. In the different moisture content groups, the fungi were predominantly *Aspergillus*, *Wallemia*, and *Ciboria* in the powder, paste, and strip groups; *Aspergillus*, *Wallemia*, and *Sporormiella* were the dominant genera in the mud-like group (Figure 2B). The overall trend was that the relative abundance decreased with increasing moisture content. Regardless of the grouping type, *Aspergillus* was the most prevalent fungal strain.

### 3.4. Diversity of Fecal Fungal Communities in Musk Deer with Different Musk Quality Groups

We next performed α diversity analysis based on the qualified sequencing depth, with a mean good coverage of 99.94% (range of 99.68–99.98%) for the fecal fungi of musk deer in different groups. To explore fungal community differences among musk deer with different qualities, the Chao1 index of the α diversity index was estimated using linear mixed models.

**Figure 3:**
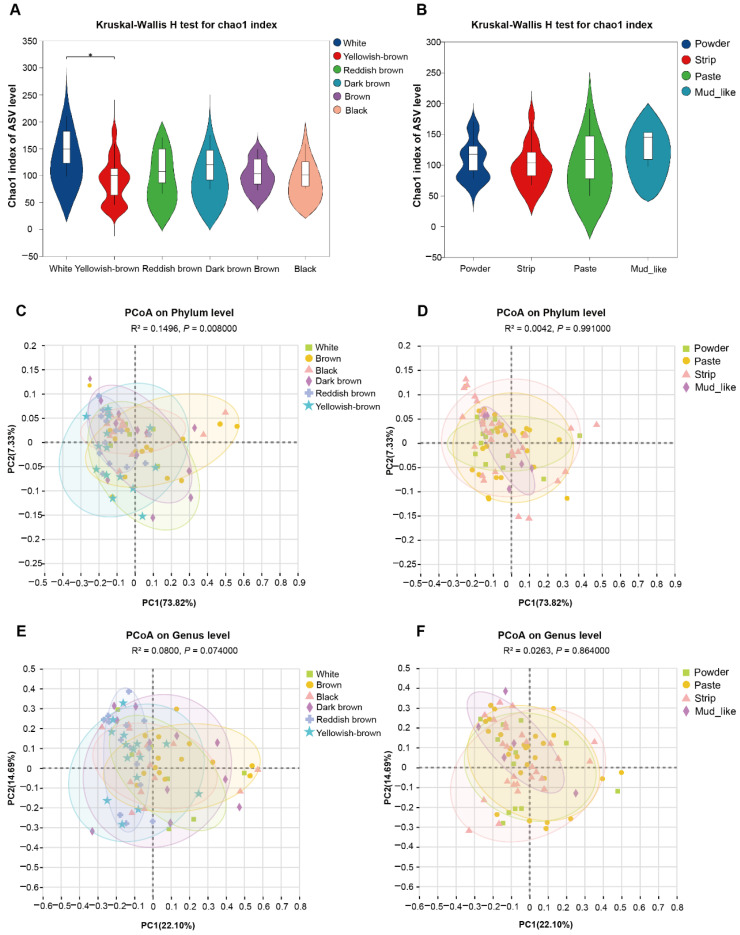
The diversity of fungal communities in musk deer with different musk qualities (* *p* < 0.05). (**A**) The species richness of different color groups. (**B**) The species richness of different moisture content groups, which were associated with the α diversity of the fecal fungi of musk deer, as measured by the Chao1 index. (**C**,**D**) PCoA of fecal fungal composition with different color and moisture contents at the phylum level, respectively. (**E**,**F**) PCoA of fecal fungal composition with different color and moisture contents at the genus level, respectively. These values are based on the weighted UniFrac distances analysis.

We can observe that the Chao1 index tended to plateau, indicating that the sequencing results are analytically reasonable (Figure S2). The results indicate that the α diversity of the fecal fungal community of musk deer with different musk colors significantly differed. The α diversity analysis of the ASVs revealed that the Chao1 diversity index significantly differed between white and yellowish-brown musk (*p* < 0.05). The overall trend manifested as a relative decrease in fungal abundance as the color deepened (Figure 3A). There were no significant differences in the Chao1 diversity indices among the groups with different moisture contents (Figure 3B). Overall, as the moisture content increased, the relative abundance of fungi also tended to increase.

To conduct subsequent analyses on the basis of various commonly used sample-to-sample distance metrics and to facilitate the observation of the degree of differences and patterns of change among the samples, principal coordinate analysis (PCoA) was employed for exploration. For the fecal fungal community, the distributions of β diversity measures (weighted UniFrac distances) were compared for the different group populations. The Adonis method was used to analyze the sample differences among the different groups, and we found that there were no differences in fungal colonies at the genus level. At the phylum level, there were differences in the β diversity among the different color groups (Figure 3C–F).

### 3.5. Forest Musk Deer Producing Normal Musk Have More Stable Fecal Fungal Communities

**Figure 4:**
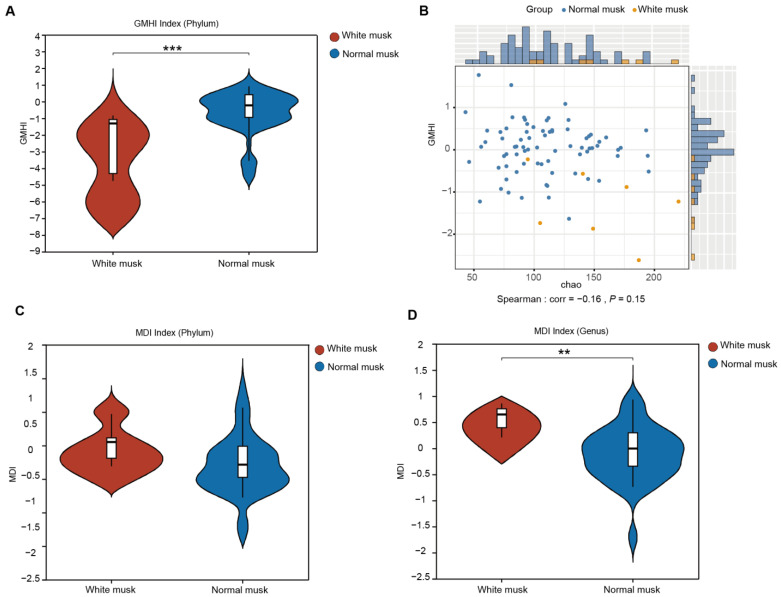
The GMHI and MDI between normal and white musk. (**A**) The GMHI between the white musk group and the normal musk group. Each dot in the scatter plot represents an ITS sample. (**B**) The distribution of white and normal musk samples across each axis parameter. (**C**) The MDI of the white musk group at the phylum level (*p* = 0.0584). (**D**) The MDI of the white musk group at the genus level (*p* = 0.002639) (** *p* < 0.01, *** *p* < 0.001).

White musk was uniquely classified as “unhealthy” due to its distinct characteristics in captive populations. It is the only color that has been explicitly linked to poor health (chronic stress, higher disease incidence) in prior studies [10], whereas other colors (e.g., brown, black, dark brown, reddish brown, yellowish-brown purple-red, brown, and yellow), although they vary in market quality, are considered “normal” because they do not correlate with overt health issues or dysbiosis. The gut microbiota health index (GMHI) is a robust index that assesses health status on the basis of species-level taxonomic characteristics of fecal microbial samples, with a focus on determining the likelihood of illness. Compared with the fecal microbiota of musk deer producing white musk deer, the fecal microbiota of musk deer producing regular musk presented a greater GMHI at the genus level (Mann‒Whitney U test, *p* = 0.000634) (Figure 4A). By comparing the GMHI and Chao1 diversity of each sample to test their overall consistency, the GMHI did not significantly differ in stratification between the white musk deer and regular musk deer groups compared with the Chao1 index (Figure 4B).

The intestinal microbiota dysbiosis index (MDI) determines the extent of microbial ecological dysbiosis, with a higher index indicating a greater degree of microbial disorder. At the phylum level, there was no significant difference between the two groups, as indicated by a higher MDI in the white musk group than in the normal musk group (Figure 4C). At the genus level, the MDI of the white musk group was significantly greater than that of the group producing normal musk (*p* = 0.0026) (Figure 4D), suggesting that individuals producing white musk may have some degree of fecal microbial imbalance, which may lead to disease.

### 3.6. Differential Fungi Among the Different Musk Colors and Moisture Content Groups

**Figure 5:**
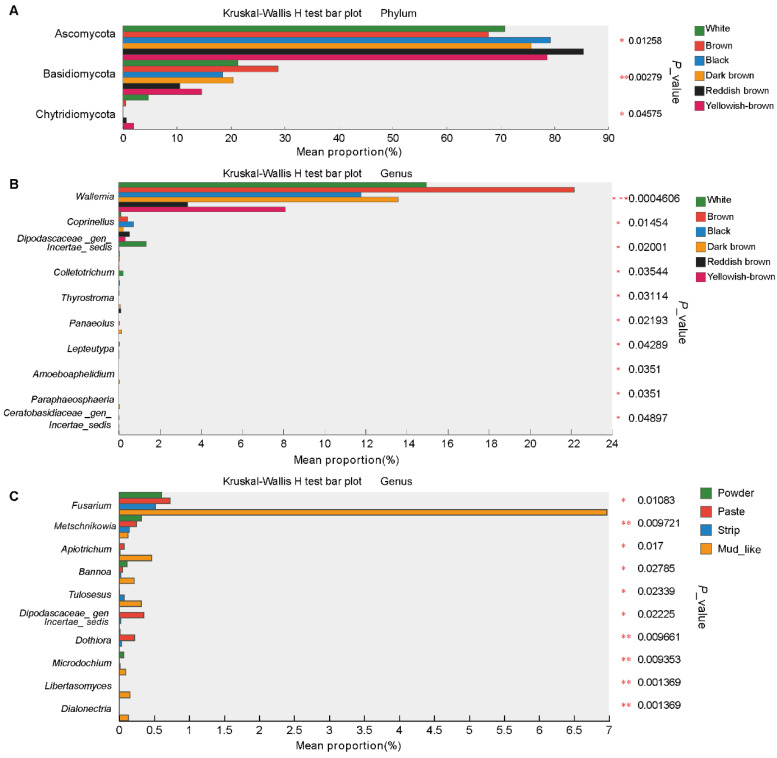
The community differences in musk with different qualities. (**A**) The composition differences within the color groups at the phylum level. (**B**,**C**) The composition differences within the color and moisture content groups at the genus levels, respectively. Data were compared via the Kruskal‒Wallis H test. (* *p* < 0.0, ** *p* < 0.01, *** *p* < 0.001).

To investigate the differences in fecal fungi within each group, we investigated the fungi across the feces of forest musk deer via the Kruskal‒Wallis H test. At the phylum level, this analysis revealed three fungi, Ascomycota, Basidiomycota, and Chytridiomycota, that differed among the fecal of forest musk deer with different color musk. The abundance of Ascomycota was significantly greater in the reddish-brown group than in the brown group, whereas the opposite was true for Basidiomycota, and the abundance of Chytridiomycota was significantly greater in the white group than in the black group. There were no differences in fecal fungi among the moisture content groups (Figure 5A).

The analysis revealed differences in fecal samples grouped by color at the genus level.The abundance of *Dipodascaceae_gen_Incertae_sedis*, *Colletotrichum*, *Lepteutypa*, and *Ceratobasidiaceae_gen_Incertae_sedis* was significantly greater in the white group than in the other groups; the abundance of *Wallemia* was significantly greater in the brown group than in the reddish brown group; the abundance of *Coprinellus* was significantly greater in the black group than in the other groups; and the abundances of *Thyrostroma*, *Panaeolus*, *Amoeboaphelidium*, and *Paraphaeosphaeria* were significantly greater in the dark brown group than in the other groups (Figure 5B). Ten fungal genera differed with respect to moisture content. The abundance of *Metschnikowia* was greater in the powder group than in the other groups, whereas the abundances of *Dipodascaceae*_ *gen_incertae_sedis,* and *Dothiora* were greater in the paste group. The abundances of the remaining seven fungi, namely *Fusarium*, *Apiotrichum*, *Bannoa*, *Tulosesus*, *Microdochium*, *Dialonectria*, and *Libertasomyces,* in the mud-like group were significantly greater than those in the other groups (Figure 5C).

### 3.7. Relationships Between Musk Production and Fecal Fungi

**Figure 6:**
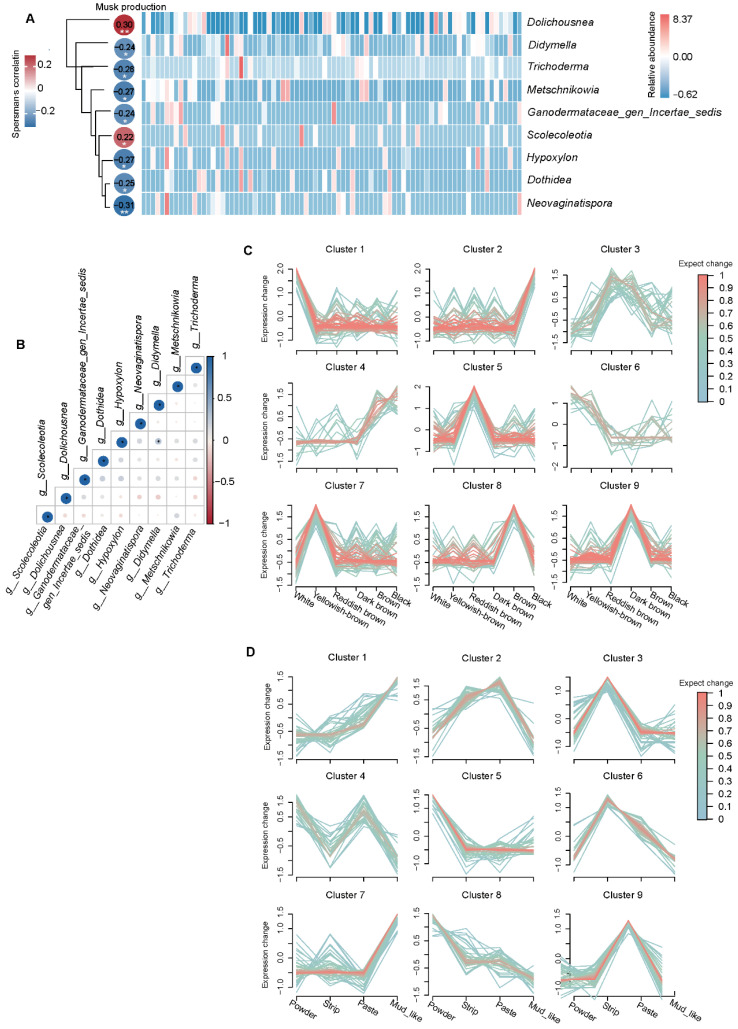
The correlation analysis and relative expression profiles. Correlation analysis was conducted between musk production and microorganisms with a prevalence rate greater than 15%, and an abundance heatmap of 9 genera related to musk production in 83 forest musk deer was generated (**A**). The correlations among microorganisms (**B**) were studied (* *p* < 0.0, ** *p* < 0.01). The dynamic expression landscape of fecal fungi from different groups was analyzed using fuzzy clustering, resulting in nine clusters of expression data. The red lines correspond to fungi with high membership values.The *y*-axis represents the standardized expression values in the Mfuzz results (**C**,**D**).

To investigate the relationship between musk production (Supplementary Table S1) and the fecal microbiota in musk deer, we constructed a correlation analysis heatmap to identify fungi associated with musk production. Fungi that appeared in more than 15% of the samples were selected for analysis. In the actual process of collecting musk during the maturation season, only normal musk is usually collected, and white musk is not completely collected. Without white musk, there is no production. Therefore, for correlation analysis, we selected only individuals that produced normal musk and those with zero musk production for analysis. The results reveal that at the genus level, *Dolichousnea* and *Scolecoleotia* were significantly positively correlated with musk production. *Dolichousnea* and *Scolecoleotia* were detected in 28% and 19% of the samples, respectively. Spearman’s correlations with musk production were *r* = 0.29 (*p* = 0.006) for *Dolichousnea* and *r* = 0.21 (*p* = 0.048) for *Scolecoleotia* (Figure 6A and Supplementary Table S4), making them the strongest positive correlates among the 927 analyzed genera.

Additionally, we investigated the relationships among these microorganisms and observed that *Didymella* and *Dothidea*, two types of fungi, were significantly positively correlated. Seven types of fungi, namely *Metschnikowia*, *Ganodermataceae_gen_Incertae_sedis*, *Hypoxylon*, *Neovaginatispora*, *Didymella*, *Dothidea*, and *Trichoderma*, were significantly negatively correlated with musk production (Figure 6A and Supplementary Table S4).We speculate that Didymella and Dothidea may play a certain regulatory role within the host, working together to regulate the host’s intestinal health and affecting the production of musk (Figure 6B).

The relative expression profiles of fungi with respect to different musk qualities revealed the expression patterns of different colors and water contents. In white musk, Cluster 1 and Cluster 6 microorganisms were highly expressed, and the three genera with the greatest relative abundance were *g__Dolichousnea*, *g__Synchytrium*, and *g__Papiliotrema* (Figure 6C). In the mud-like musk, microorganisms in Clusters 1 and 7 were highly expressed, and the top three in terms of relative abundance were *g__Sporormiella*, *g__Cladosporium*, and *g__Dolichousnea.* These results reveal that *g__Dolichousnea* was present in the intestines of some musk-producing musk deer; therefore, we speculate that this fungus may have a certain regulatory effect on the bodies of musk deer (Figure 6D).

### 3.8. Predicted Functions of the Fecal Fungal Community of Musk Deer

**Figure 7:**
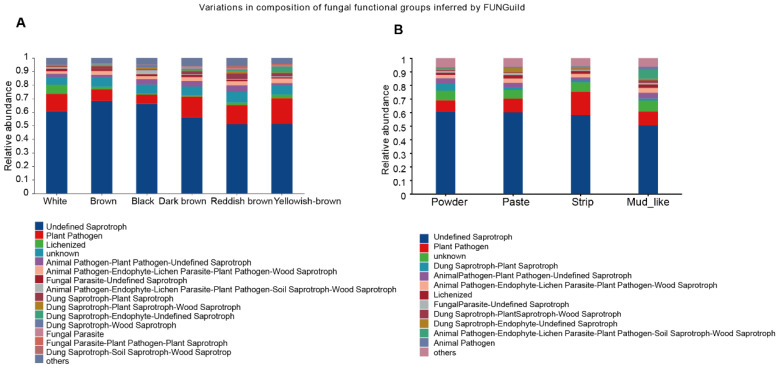
The potential biological function of fungi for musk with different colors (**A**) and moisture contents (**B**).

To predict the functions of the fecal fungal community, we employed FUNGuild (Fungi Functional Guild) to construct a database linking fungal taxonomy with functional guilds. By using this database for the functional classification of fungi, we found that there was not much difference in the enrichment of microbial functions among the different groups, with the two main functions concentrated in Undefined Saprotroph and Plant Pathogen. In addition, we found significant functional differences across the color groups, with Lichenized dominating in white musk, Animal Pathogen–Plant Pathogen–Undefined Saprotroph and Dung Saprotroph–Plant Saprotroph being predominant in red‒brown musk (Figure 7A,B). Our analysis revealed significant functional disparities among the distinct moisture content groups, with Dung Saprotroph–Plant Saprotroph leading in powdered musk, Dung Saprotroph–Endophyte-Undefined Saprotroph dominating in creamy musk, and Animal Pathogen–Endophyte–Lichen Parasite–Plant Pathogen–Soil Saprotroph–Wood Saprotroph holding a dominant position in mud-like musk.

## 4. Discussion

Within the host organism, there exists a complex interplay between the microbial community and the host, which includes mutualistic symbiosis, commensalism, and even potential pathogenic relationships, depending on the type of microorganisms, the host’s physiological state, and environmental factors [41]. Within this ecosystem, fecal fungi and other microorganisms synergistically contribute to the stability of the fecal microbiota [42,43]. Recent studies have highlighted the probiotic role on nutrient digestion, collaboration with bacteria to regulate intestinal health, and enhancement of the host immune system [44]. Moreover, several studies have implicated fecal fungi in immune regulation and lipid metabolism [45,46]. Nevertheless, the impact of fecal fungi on musk production and quality is an area that has yet to be fully explored. In this study, we focused on musk deer and collected 89 fresh fecal samples, supplemented with data on musk production and quality metrics, such as color variation and moisture content. Through ITS sequencing of the fecal samples, we conducted a correlation analysis to elucidate the relationships between fungus and musk production and quality.

### 4.1. Dominant Fungal Communities in Musk

According to our species annotation findings, the two fungal phyla Ascomycota and Basidiomycota were the absolute dominant phyla across the various groups, accounting for a significant 88.55% of the total composition. This result is similar to that of most other animal studies [47]. These fungi can assist in nutrient uptake by facilitating the decomposition of cellulose, hemicellulose, and lignin in food [48,49]. The secondary metabolites they produce, such as extracellular polysaccharides, exhibit antioxidant, immunostimulatory, anti-tumor, and antibacterial properties [50]. Furthermore, the three fungal genera *Aspergillus*, *Wallemia*, and *Ciboria* were overwhelmingly dominant in most of the subgroups, comprising up to 52.49% of the microbial community. These fungi are significantly involved in lipid and amino acid metabolism. They also have an inverse relationship with the expression of certain genes associated with inflammation, playing a role in preserving the stability of the fecal microbiome [51]. This indicates that these fungal communities of forest musk deer might be responsible for assisting in food digestion. Fatty acids are probably absorbed and transformed by the fecal fungi to facilitate the synthesis of musk [52].

### 4.2. Pathogenic Fungi Related to Abnormal Musk

Studies have shown that the amino acid and hormone contents in white musk are significantly lower than those in normal musk, whereas the fat content is greater than that in normal musk. Abnormal musk may serve as an indicator of the health status of forest musk deer [10]. GMHI and MDI were used to investigated the intestinal health and dysbiosis indices of musk deer producing white musk. White musk-producing deer showed higher MDI and lower GMHI, indicative of an imbalance in the composition and function of the intestinal microbiota, such as chronic gut inflammation or stress-related disorders. As a well-known plant pathogen, once plants infected by *Colletotrichum* are consumed by animals, the secondary metabolites produced in the feces can pose a risk to animal health [53]. Li et al. reported that the intake of tea beverages containing *Colletotrichum* leads to increased fat content and certain types of kidney damage in mice [54]. It is also increasingly reported to cause ophthalmic infections in humans [55]. Mud-like musk groups harbored more fungi, such as *Fusarium*, *Apiotrichum*, and *Sporormiella*. These fungi are all pathogenic to some extent and are harmful fungi commonly found in food and feed. The toxins they produce pose a threat to the health of animals [56,57,58]. For example, *Fusarium* is a conditionally pathogenic filamentous fungus that can cause invasive or localized infections and mycotic keratitis in humans [59,60]. *Fusarium camptoceras* was reported to cause food rot and tail decay in cattle [61]. *Apiotrichum mycotoxinivorans* (originally known as *Trichosporon mycotoxinivorans*) can cause chronic lung infection [62]. *Sporormiella* can strongly inhibit cholesterol synthesis in human liver cells and has antifungal activity against *Candida albicans* and *Aspergillus fumigatus* [63].

Among the fungi that caused GMHI and MDI imbalance in our study, only *Fusarium camptoceras* is an ungulate pathogenic fungus [61], although many fungi were animal-associated pathogens [64]. *Fusarium,* dominating in mud-like musk, is a known mycotoxin producer that may induce subclinical toxicity, affecting liver or kidney function [65] and indirectly compromising musk quality. While direct disease manifestations (e.g., diarrhea, abscesses) were not explicitly measured in this study, previous reports have linked similar fungal pathogens to gastrointestinal distress and immune suppression in livestock [66], which likely extend to musk deer. These fungi thrive in dysbiotic environments, suggesting they may behave as latent pathogens rather than directly causing overt disease in musk deer. However, their presence as dominant taxa in low-quality musk groups (e.g., white and mud-like musk) indicates that they contribute to intestinal dysfunction, which is a key driver of abnormal musk formation. However, research on the pathogenicity of these opportunistic pathogenic fungi in musk deer is still scarce, and the specific influential mechanism requires subsequent studies.

There are many reasons for the presence of pathogenic fungi in the intestines of musk deer, including poor feeding and management hygiene conditions, low-quality feed, the stress response of musk deer, and horizontal transmission through direct contact with infected individuals or indirect exposure to contaminated water/food. In response to these possible pathogenic factors, a multi-faced approach can be applied in musk deer captivity, such as enhanced hygiene and environmental management, probiotic interventions, immunomodulatory strategies and dietary optimization.

### 4.3. Beneficial Fungi Facilitating Musk Production

In addition, we conducted a correlation analysis between the annual musk production of forest musk deer and the fecal microbiota of forest musk deer. Two fungal genera, *Dolichousnea* and *Scolecoleotia*, were significantly positively correlated with musk production. Other studies have also shown that *Dolichousnea* can be used as a medicine to treat animal diseases [67]. We found that the relative abundance of this fungus is also relatively high in the feces of some musk deer that produce abnormal musk. We speculate that this represents self-healing in musk deer, where the stability of the intestinal microbiota is self-regulated to restore health. *Scolecoleotia*’s association with efficient cellulose degradation (common in ruminant gut fungi) may enhance nutrient absorption, indirectly supporting musk gland biosynthesis [48]. Beneficial fungi positively correlated with musk production (*Dolichousnea*, *Scolecoleotia*) may be used to develop probiotic supplements, restoring gut microbiota balance and suppressing pathogens. These two fungi can even be used as candidate microorganisms for further functional studies to increase musk production.

## 5. Conclusions

When we collected musk during the musk maturation season, we found musk with different apparent characteristics, including different colors and water contents. In recent years, many studies have shown that microorganisms play a very important role in nature. This study is the first to analyze the impact of the fecal fungi of forest musk deer on musk production and quality by collecting the feces of forest musk deer producing musk with different characteristics. We observed a significant number of pathogenic fungi in the fecal microbiota of deer producing white musk and mud-like musk. The indices of fecal fungal health and dysbiosis for white musk and normal musk further suggest that one likely cause of the production of abnormal musk could be the disturbance of fecal fungi. The production of white musk and some types of abnormal musk may indicate the health status of forest musk deer. In addition, we found a positive correlation between *Dolichousnea* and *Scolecoleotia* fungi and musk production. In future research, we can use these two fungi as beneficial species to improve the intestinal health of musk deer, promote musk production, and develop probiotics. This provides high-priority hypotheses for mechanistic research in further research. This exploratory work is essential for advancing the field, as no prior studies existed on the effects of gut fungi on musk deer and musk traits.

We carried out a foundational cross-sectional analysis in this study. However, single-time point sampling has inherent limitations in capturing the temporal microbial contributions to the prolonged musk formation process. Future studies should perform longitudinal fecal sampling across musk secretion and validate whether chronic gut fungal colonization influences glandular biosynthesis over time. Multi-farm comparative studies, controlled environmental trials, and the integration of ecological factors should be taken into consideration as well. While our study has established associations between fecal fungal communities (e.g., *Dolichousnea*) and musk quality/production traits, we did not experimentally test whether these fungi directly influence musk synthesis pathways (e.g., steroid hormone biosynthesis, lipid metabolism), and it is important to note that correlational findings do not confirm causal mechanisms. Future studies can be carried out from the following perspectives: (1) Validate causal relationships by co-culturing gut fungi with musk gland cells or using musk rats as models to test whether *Dolichousnea* or *Scolecoleotia* enhance musk precursor synthesis. (2) Investigate fungi–bacteria interactions using metagenomics, identifying shared metabolic pathways (e.g., steroid hormone biosynthesis, lipid metabolism) that drive lipid derivative production in both the gut and musk gland. (3) Isotope-labeled fungal metabolites should be traced to track whether gut-derived compounds enter systemic circulation or directly impact glandular cells, leaving their role in musk synthesis speculative. (4) Translate correlational findings into applications by formulating probiotics containing *Dolichousnea* and prebiotics enhancing its growth, combined with dietary adjustments to optimize gut fungal metabolism for improved musk quality.

## Data Availability

The original contributions presented in this study are included in the article. Further inquiries can be directed to the corresponding authors.

## Supplementary Materials

The following supporting information can be downloaded at https://www.mdpi.com/article/10.3390/ani15111622/s1: Figure S1: The relationship between musk production and the age of forest musk deer; Figure S2: Rarefaction curves reflecting the diversity of Chao1 from the fecal of musk deer analyzed in this study; Table S1: Musk sample and musk production group information; Table S2: Musk moisture content; Table S3: Basic information concerning musk deer feces sequencing results; Table S4: Correlation analysis between musk production and fecal microbiota.
