# Supplementary material for: An Attempted Correlation Between the Fecal Microbial Community of Chinese Forest Musk Deer (Moschus berezovskii) and Differences in Musk Production and Quality
Source: Animals (Basel). 2025 May 31;15(11):1622. doi: 10.3390/ani15111622 (PMC12153584; doi:10.3390/ani15111622)
Supplement: Supplementary file 1 [file animals-15-01622-s001.zip › animals-3591348-supplementary figures.pdf]

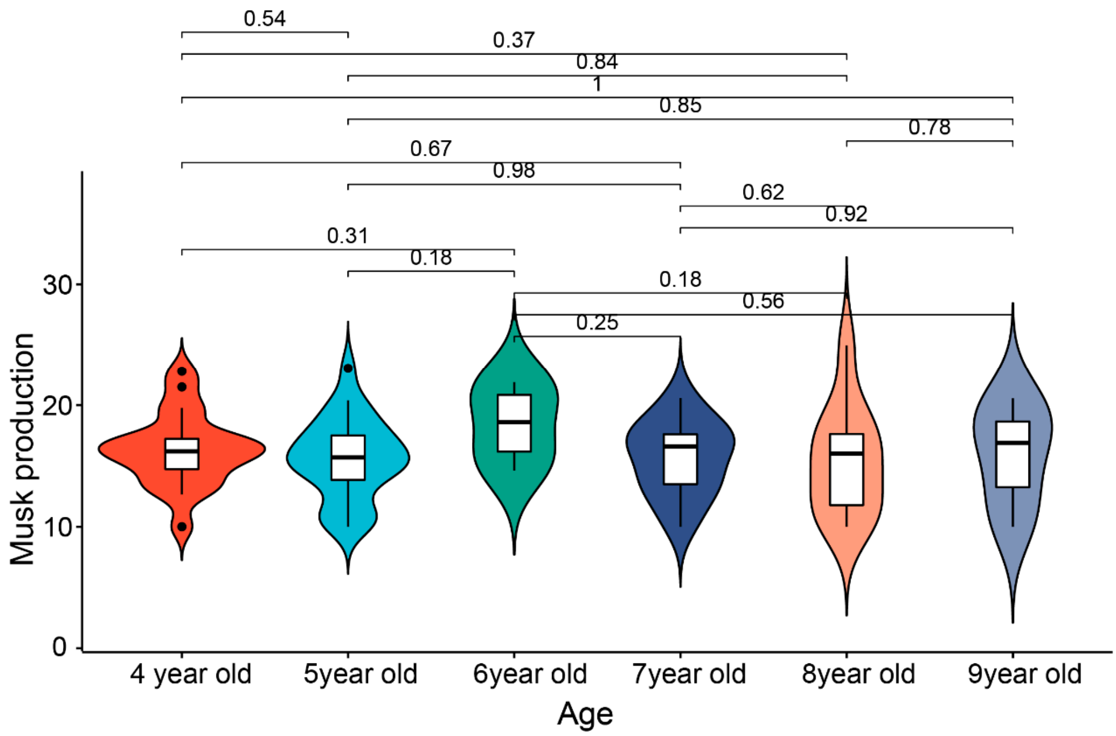

Figure S1. The relationship between musk production and age.

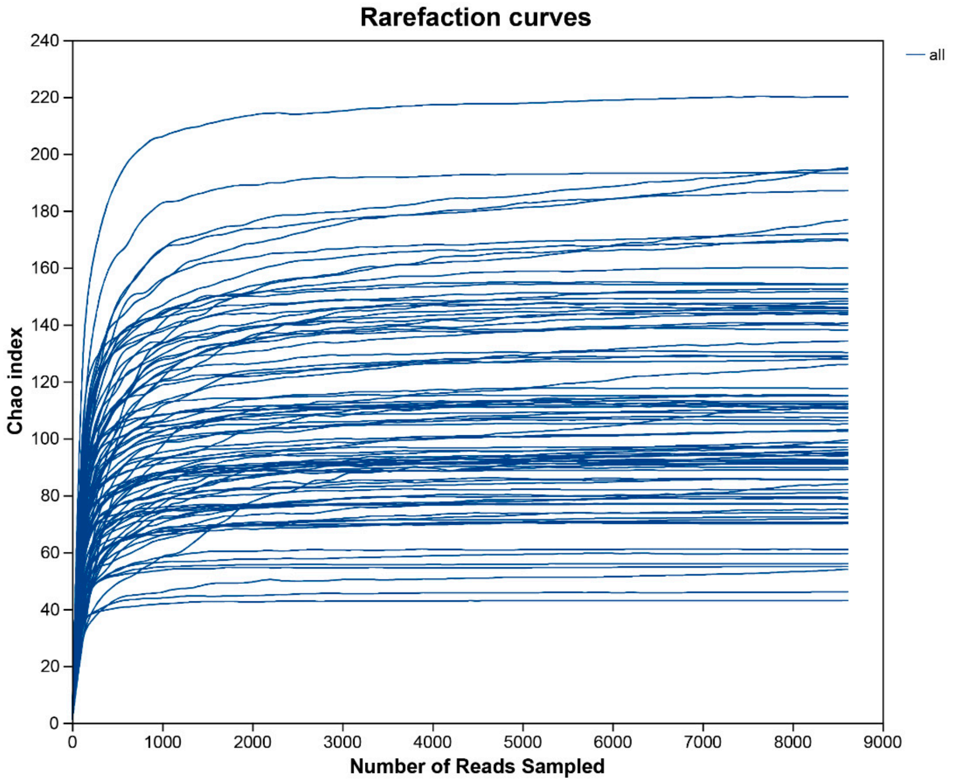

Figure S2. Rarefaction curves reflecting the diversity of fungal ASVs from the feces of musk deer as analyzed in this study.
